# Early T-Cell Precursor Leukemia Has a Higher Risk of Induction-Related Infection among T-Cell Acute Lymphoblastic Leukemia in Adult

**DOI:** 10.1155/2020/8867760

**Published:** 2020-12-24

**Authors:** Kangyu Huang, Min Dai, Qiuli Li, Nannan Liu, Dainan Lin, Qiang Wang, Xuan Zhou, Zhixiang Wang, Ya Gao, Hua Jin, Xiaoli Liu, Qifa Liu, Hongsheng Zhou

**Affiliations:** ^1^Department of Hematology, Nanfang Hospital, Southern Medical University, Rd 1838 North Guangzhou Avenue, Guangzhou 510515, China; ^2^Department of Hematology, The People's Hospital of Guangxi Zhuang Autonomous Region, Nanning, China

## Abstract

**Background:**

Infections are an important cause of morbidity and mortality for acute lymphoblastic leukemia (ALL). However, the reports regarding risk factors of induction-related infection are roughly unknown/limited in adult T-ALL during induction chemotherapy.

**Methods:**

We performed a retrospective cohort study for the prevalence and risk predictors of induction-related infection among consecutive T-ALL patients (*N* = 97) enrolled in a PDT-ALL-LBL clinical trial. Of 97 patients with T-ALL enrolled in the trial, 46 were early T-cell precursor (ETP) ALL and 51 were non-ETP ALL.

**Results:**

When compared with non-ETP, ETP ALL subtype was characterized with lower neutrophil count (1.35 × 10^9^/L vs. 8.7 × 10^9^/L, *P* < 0.001) and lower myeloid percentage in the bone marrow (13.35% vs. 35.31%, *P* = 0.007). Additionally, ETP ALL had longer neutropenia before diagnosis (*P* < 0.001), as well as during induction chemotherapy (*P* < 0.001). Notably, the ETP cohort experienced higher cumulative incidence of clinically documented infections (CDI; 33.33%, *P* = 0.001), microbiologically documented infections (MDI; 45.24%, *P* = 0.006), resistant infection (11.9%, *P* = 0.013), and mixed infection (21.43%, *P* = 0.003), respectively, than those of the non-ETP cohort. Furthermore, multivariable analysis revealed that T-ALL mixed infection was more likely related to chemotherapy response (OR, 0.025; 95% CI 0.127-0.64; *P* = 0.012) and identified myeloid percentage as a predictor associated with ETP-ALL mixed infection (OR, 0.915; 95% CI 0.843-0.993; *P* = 0.033), with ROC-defined cut-off value of 2.24% in ETP cohorts.

**Conclusions:**

Our data for the first time demonstrated that ETP-ALL characterized with impaired myelopoiesis were more susceptible to induction-related infection among T-ALL populations.

## 1. Introduction

Infections are important causes on morbidity and mortality of children and adults with acute leukemia [1–7]. These patients are at a high risk of infection, particularly in induction therapy, likely related to the intensive therapy resulting in profound neutropenia and lymphopenia. [1–3] Infections contribute to not only mortality but also prolonged hospitalization, compromised chemotherapy delivery, and increased health care utilization. Furthermore, empiric and preemptive treatments with broad-spectrum antibiotics may lead to the emergence of multidrug resistance microbiological flora.

Although it is well known that invasive infections are common, little is known about predictors of infections in acute lymphoblastic leukemia (ALL). A report from the Medical Research Council in UK found that Down syndrome was the most significant risk factor for infection-related mortality (IRM) of childhood ALL [1]. Recently, early T-cell precursor ALL (ETP-ALL) was recognized as a form of T-cell ALL with poor prognosis [8–10]. ETP-ALL is characterized with a unique immunophenotype of early arrest in T-cell differentiation, accompanied by markers of stem cells and myeloid progenitors [11–17]. It is less documented about the epidemiology and risk factors of invasive infection in adult ALL, particularly in ETP-ALL, undergoing induction chemotherapy. [18, 19]

To address the current lack of clinical evidence, we performed a retrospective cohort analysis based on a consecutive T-ALL patient trial enrolled onto pediatric-inspired, PEG-L-asparaginase-intensified, and MRD-directed precision-classification-directed-target-total-therapy (PDT-ALL-LBL). The objectives of this study were to explore the incidence of morbidity and mortality, the relationship of disease-related variables with infections, and potential clinical risk factors.

## 2. Patients and Methods

### 2.1. Patients and Induction Chemotherapy

A total of 97 adult T-ALL patients were enrolled in pediatric-inspired, PEG-L-asparaginase-intensified, and MRD-directed PDT-ALL-LBL protocol (Clinical Trials ID: NCT03564704). The study was conducted in accordance with the Declaration of Helsinki and approved by Institutional Review Board (IRB)/Ethical Committee (EC) according to institutional guidelines. ETP patients were diagnosed by WHO immunophenotypic criteria: [1] absent (<5% positive cells) CD1a, CD8 expression; [2] absent or dim (<75% positive cells) CD5 expression; and [3] expression (>25% positive cells) of 1 or more myeloid (CD11b, CD13, CD33, and CD117) or stem cell (CD34, HLA-DR) markers [20]. An intensive chemotherapy regimen for induction consists of VICLD (vincristine/VCR: 1.4 mg/m^2^ for day 1, 8, 15, and 22; idarubicin/IDA: 10 mg/m^2^ for day 1, 8; cyclophosphamide/CTX:1 g/m^2^ for day 1; pegaspargase/PEG-asp: 2000 IU/m^2^ for day 1, 15; dexamethasone: day 1 to 24 as continuous infusion; and chidamide: 10 mg from day 1 to 24) (Supplemental Table 1). Bone marrow test and flow-based MRD-assessment were performed on day 15, day 24, and day 45 during the induction phase. The definitions of infections were based on the European Organization for Research and Treatment of Cancer (Supplemental Table 2) [21]. Mixed infection was defined as more than one microbiologically documented infection during induction therapy. Episodes of infections recorded during induction therapy within a time span of 24 days were analytically reviewed. Echinocandins, a class of antifungal drugs used for prophylaxis, were administered during neutropenia during the induction phase. Patients received no prophylaxis for antibacterial. Infection-related mortality (IRM) was defined by any CDI/MDI-related death during the induction treatment. Myeloid percentage in the bone marrow was determined by flow cytometry and aspirate smear by Wright-Giema stain on bone marrow samples at diagnosis and measured as the mean plus/minus the standard deviation.

### 2.2. Potential Predictors

Potential predictors of infection complications included age, gender, leukemia subtype (ETP, non-ETP), immunophenotype (CD4, CD8, CD13, CD33, CD34, CD38, CD11b, CD65, and HLA-DR), WBC count (0-<4, 4-30, and >30 × 10^9^/L), neutrophil count (0-<0.5, 0.5-1.5, and >1.5 × 10^9^/L), LDH level (<600, ≥600 U/L), duration of neutropenia, and myeloid-percentage in the bone marrow at diagnosis.

### 2.3. Statistical Analysis

Associations and distinctions among clinical factors, including age, gender, leukemia subtype, immunotype (CD4,CD8,CD13, CD33, CD34, CD38,CD11b, CD65, and HLA-DR), WBC(0-<4, 4-30, and >30 × 10^9^/L), neutrophil (0-<0.5, 0.5-1.5, and >1.5 × 10^9^/L), LDH(<600, ≥600 U/L), duration of neutropenia, and myeloid percentage in the bone marrow at diagnosis and infection events were evaluated. Data were analyzed by using SPSS 20. Variables were compared with the *χ*-squared test or Fisher's exact test and continuous variables with an appropriate nonparametric test. The relationships between the potential predictors and binary outcome, sepsis death, and other variables were examined using multivariate logistic regression. A *P* value (two tailed) of < 0.05 was statistically significant.

## 3. Results

### 3.1. Study Diagram of Induction-Related Infections

**Figure 1:**
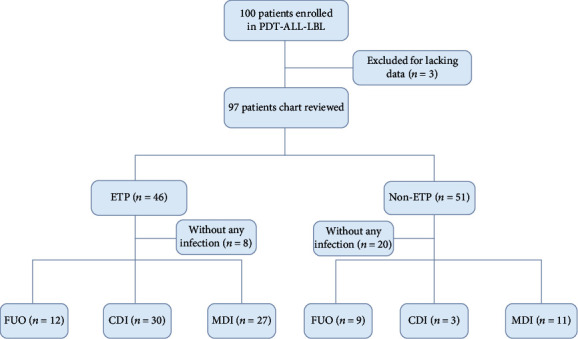
Flow diagram of induction-related infections. Forty-six patients in the ETP cohort experienced 69 episodes of induction-related infection, while 51 cases in non-ETP cohort had 23 episodes of infections in 24 days of induction phase. ETP: early T-cell progenitor ALL; non-ETP: non-early T-cell progenitor ALL; FOU: fever of uncertain origin; CDI: clinically documented infection; MDI: microbiologically documented infection.

From Feb 2016 to Dec 2019, 100 adult patients with newly diagnosed T-ALL were enrolled in this study. Three patients were excluded for lacking clinical raw data as shown in Figure 1. A total of 97 patients received intensive induction chemotherapy according to the PDT-ALL-LBL protocol and experienced 92 episodes of infections within first 24 days of induction phase (Figure 1). As shown in Figure 1, induction-related infections included fever of uncertain origin (FOU) 22.83% (21/92), 35.87% (33/92) clinically documented infections (CDI), and 41.3% (38/92) microbiologically documented infections (MDI). Noteworthily, the majority of FOU (57.14%), CDI (90.91%), and MDI (71.05%) occurred in ETP cohort (Figure 1).

### 3.2. Clinical Characteristics

**Figure 2:**
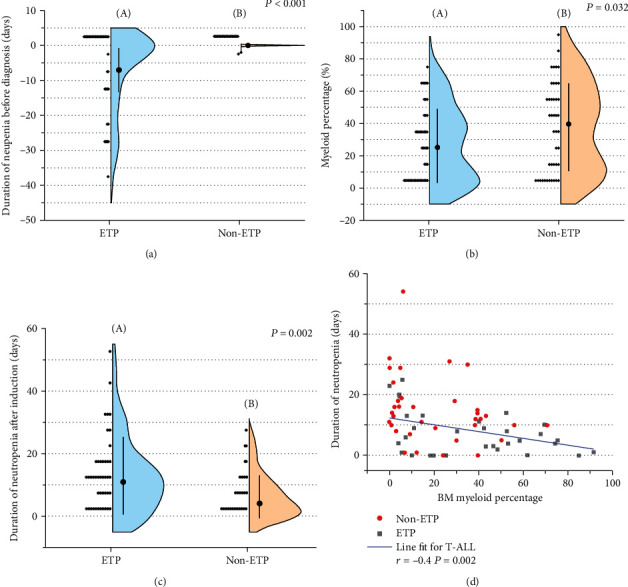
Clinical characteristics of the ETP and non-ETP cohorts. (a) Comparison of neutropenia at prediagnosis between the ETP (A) and non-ETP (B) patients. (b) Analysis of myeloid percentage in the bone marrow in the ETP (A) and non-ETP (B) cohorts. (c) Comparison of neutropenia duration during induction in the ETP (A) and non-ETP (B). (d) Linear relationship analysis between the myeloid percentage and neutropenia duration during induction, ETP is shown as red-dot and non-ETP as solid black square. Quantitative variables are expressed as mean (a) or median (b, c).

Overall, 46 out of 97 patients enrolled in the PDT-ALL-LBL trial were diagnosed as ETP ALL. Basic clinical characteristics are presented in Table 1. When compared with non-ETP, ETP patients had lower WBC, neutrophil, and LDH (*P* < 0.001) and higher platelet count (*P* < 0.001). Notably, myeloid percentage at diagnosis in ETP leukemia was lower than that of non-ETP ALL (median, 13.35% vs. 35.31%, *P* = 0.007). ETP cohort presented a higher incidence (34.78% vs. 1.96%) and longer duration (18 days vs. 1 day) of neutropenia at prediagnosis phase and during induction chemotherapy (Figures 2(a)–2(c)). A negative linear relationship was identified between the duration of neutropenia in induction treatment and BM myeloid percentage (*r* = −0.4, *P* < 0.001) in the T-ALL group (Figure 2(d)), but there was no relationship of this kind observed in the ETP and non-ETP cohorts. As shown in Figure 2(d), most cases with ETP were located above the line fitting, while non-ETP were below the line, indicating more impaired myelopoiesis in the ETP cohort than that in non-ETP ALL. The CR rate after first induction treatment in patients with ETP was significantly lower than that of non-ETP patients (*P* = 0.001) as expected. However, the death rate during induction treatment between the ETP and non-ETP patients was similar (*P* = 0.593, Table 1).

### 3.3. Clinical-Biological Features of Induction-Related Infections

**Figure 3:**
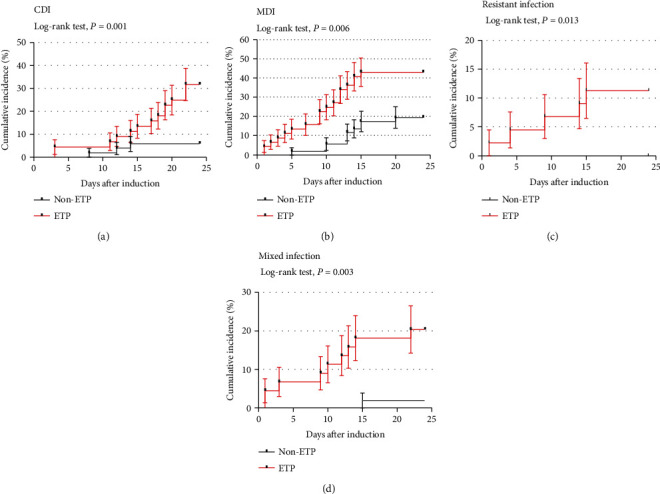
Kaplan-Meier analysis of induction-related infections in the ETP and non-ETP cohort.

During the first 24 days of intensive treatment, 97 patients experienced a total of 92 infectious episodes, consisting of 21 episodes of fever of unknown origin (FUO, 22.83%), 33 episodes of CDI (35.87%), and 38 episodes of MDI (41.3%). Firstly, we compared the CDI and MDI between the ETP and non-ETP cohorts. Log-rank tests demonstrated that the ETP cohort had a higher cumulative incidence of both CDI (30.43% vs. 5.88%, *P* = 0.001) and MDI (41.3% vs. 19.6%, *P* = 0.006), compared with the non-ETP cohort. In terms of pathogens, we further analyzed infections caused by Gram-negative and Gram-positive organisms and fungi between the ETP and non-ETP cohorts and found that there was no significant difference observed (Supplemental Figure 1). On the other hand, the ETP cohort had a higher incidence of drug resistant (10.87% vs. 0%, *P* = 0.013) and mixed infection (19.57% vs. 1.96%, *P* = 0.003) (Figure 3).

Cumulative incidence with first 24 days as indicated as incidence rate plus or minus two standard errors. Resistant infection: Diagnosed resistant bacterial infection with supportive microbiological evidence, such as a positive culture, antigen or PCR test results, or characteristic histopathological findings. Mixed infection: Identified more than one microbiologically documented infection during the first induction therapy [21].

Next, we analyzed infection sites and pathogen types in induction-related infection. As shown in Table 2, bloodstream and pulmonary infections were the most prominent sites of infection in the T-ALL group. Of note, both the bloodstream and pulmonary infections were more common in the ETP cohort than in the non-ETP cohort (Table 2(a), *P* = 0.057, *P* = 0.01). In the context of disseminated infections, 19 cases occurred in the ETP-ALL cohort and only 4 cases in the non-ETP cohort. The fascia, perianal, intestinal tract, endocardium, and bone marrow were involved in infections of the ETP cohort, while limited sites in the skin and gastrointestinal tract in the non-ETP cohort (Table 2(a)). Therefore, an increased awareness of disseminated infection potential for ETP patients is required during the induction period. Actually, infection sites of ETP ALL are also more scattered than the non-ETP patients in the prediagnosis phase and clinical lesions involved in the gastrointestinal tract, central nervous system, cardiovascular system, skin, and soft tissue (Supplemental Table 3).

Besides, we further reviewed the pathogens of MDI in the ETP and non-ETP cohorts. As shown previously, the ETP cohort presented a markedly higher incidence of MDI than non-ETP. Gram-negative bacteria (26/38, 68.42%) predominated among the pathogens were isolated from both cohorts, followed by fungi (6/38, 15.79%) and Gram-positive bacteria (6/38, 15.79%). *Klebsiella* spp. and *Pseudomonas aeruginosa* accounted for most Gram-negative infections (Table 2(b)). Nonfermenting Gram-negative bacilli is frequently related to intrinsic antibiotic resistance. In our study, 7 of 9 nonfermenting bacterial episodes, including *Acinetobacter* spp., *Stenotrophomonas maltophilia*, and *Sphingomonas paucimobilis*, were detected in only the ETP cohort. Particularly, 10 out of 97 patients (10.31%) were infected with more than one pathogen, including 9 ETP and 1 non-ETP patients (OR 9.978; 95% CI 1.314-75.761; *P* = 0.005), indicating that the ETP cohort had a higher risk of mixed infection during induction chemotherapy. Furthermore, antimicrobial resistance was only found in 5 ETP patients (5/46, 10.87%), including carbapenem-resistant *Klebsiella pneumoniae* (CRKP), pan-drug-resistant *Acinetobacter baumanni* (PDRAB), methicillin-resistant *Staphylococcus aureus*- (MRSA-) resistant Aeromonas hydrophila and ESBLs-producing *Escherichia coli* (Supplemental Table 4).

Taken together, our data showed that ETP leukemia was associated with a higher incidence of CDI and MDI, and severe and complicated infections, compared with the non-ETP subset.

### 3.4. Infection-Related Mortality (IRM)

In our study, IRMs of induction therapy were 7.2% (7/97) in T-ALL, and 8.7% (4/46) in the ETP cohort and 5.88% (3/51) in the non-ETP cohort, respectively. Four patients with ETP ALL died of severe and complicated infections induced by CRE, PDRAB, and nonfermenting bacteria (Supplemental Table 5). To identify the potential risk factor associated with IRM during induction, we performed univariate and multivariate analyses. In univariate regression analysis, Gram-negative bacteria and mixed infection were associated with higher risk of IRM in T-ALL patients (OR, 20.4; 95% CI 2.318-179.55; *P* = 0.007 and OR, 10.25; 95% CI 1.852-56.736; *P* = 0.008) (Table 3). Collectively, our study indicated that mixed infections contributed to IRM in T-ALL (data not shown) and ETP-ALL cohorts.

### 3.5. Risk Factors Associated with Infections

**Figure 4:**
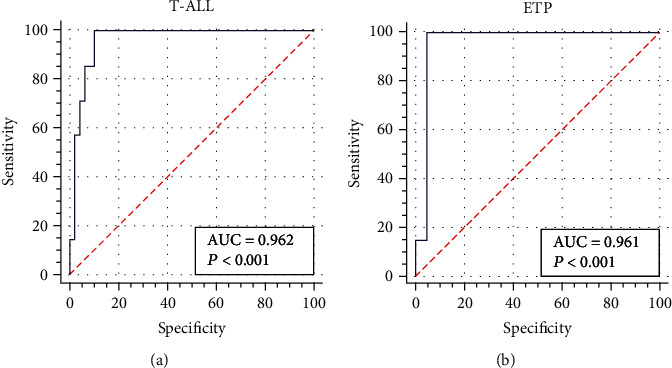
Receiver operating characteristic (ROC) curve for the multivarivate model of mixed infection in the T-ALL (a) and ETP-ALL (b) cohorts.

To identify the potential risk factors associated with induction-related infection, univariate and multivariable analyses were performed. BM myeloid percentage was related to prediagnosed infections in T-ALL (univariate model *P* = 0.003, multiple model *P* = 0.005) and ETP cohorts (univariate model *P* = 0.009, multiple model *P* = 0.011) (Supplemental Table 6). During remission induction in the first 24 days, WBC count, NEU count, phenotype, and myeloid percentage did not significantly influence the development of CDI and MDI among T-ALL/LBL, ETP, or non-ETP patients (data not shown). In terms of mixed infection in the T-ALL cohort and ETP cohort, potential clinical risk factors would be different. In univariate regression analysis, for the T-ALL group, induction treatment responses (CR or not) (*P* = 0.012) were related to mixed infection, while in the ETP cohort, myeloid percentage was the only clinical risk factor (*P* = 0.033) (Table 4). Multivariable regression analysis demonstrated that induction treatment response (CR or not) and myeloid percentage was significantly associated with mixed infection in T-ALL (*P* = 0.022, *P* = 0.035, AUC = 0.962), with an ROC defined cut-off value of 12.27%. And myeloid percentage was the only infection risk factor related to the ETP cohort (*P* = 0.043, AUC = 0.961), and ROC defined cut-off value was set as 2.24% (Figure 4). Overall, these data unearthed the relationship between impaired myelopoiesis, i.e., reduced myeloid percentage and susceptibility to serious and complicated infection during induction chemotherapy.

## 4. Discussion

In this study, we investigated the prevalence of infection-related complications during induction therapy among T-ALL population and compared the clinical features between the ETP and non-ETP cohorts. It has been documented that intensive induction chemotherapy yielded serious neutropenia and high incidence of infection in ALL, but little has been reported about risk factors implicated in T-ALL population. [9, 19, 22] Our study revealed that ETP ALL was characterized with markedly impaired myelopoiesis at diagnosis, presenting with lower neutrophil and reduced myeloid percentage, when compared with non-ETP ALL. Notably, ETP experienced a significantly higher incidence of CDI, MDI, antimicrobial resistance, and mixed infection, which were 2-10-folds higher than that in the non-ETP cohort. Furthermore, our study confirmed the association between reduced myeloid percentage and the risk of mixed infection. Additionally, identified myeloid percentage as a predictor was highly associated with mixed infection in both the T-ALL and ETP-ALL cohorts. Collectively, our data provided evidences, for the first time, about the relationship between clinical features of ETP subtype and susceptibility to infection among T-ALL population during induction chemotherapy. [14, 15, 17, 23–25]

Induction-related infections account for most of the morbidity and mortality of acute leukemia. In the present study, among 92 infection episodes occurred in T-ALL population, consisting of around 80% CDI and MDI. With the same regimen of induction chemotherapy, the ETP cohort experienced three-quarters of CDI and MDI episodes among the T-ALL cohort. Furthermore, our study demonstrated that ETP patients were significantly more vulnerable to complicated infection, compared to other T-ALL patients. In addition, among the episodes of Gram-negative bacteremia, nonfermenting Gram-negative bacilli were present in one-third of episodes and predominant in the ETP cohort (7/9). Infection with resistant bacteria, including CRE, MRSA, PDRAB, and *Aeromonas hydrophila*, were only found in the ETP cohort. These data highlighted higher susceptibility to induction-related infection in ETP leukemia, in contrast to non-ETP T-ALL, which was not well addressed yet. In our study, patients received echinocandins prophylaxis for antifungi, which might contribute to the low incidence of fungal infection and no fungus-related IRM. Antibacterial prophylaxis was still controversial in ALL, and accumulating evidences indicated that levofloxacin prophylaxis reduces odds of febrile neutropenia and bacterial infection during induction in pediatric ALL [6]. Therefore, an increased awareness of infection is required for the T-ALL and ETP patients.

Our data revealed that the clinical features and chemotherapy responses might contribute to the increased risk of infection during induction chemotherapy in T-ALL. Regarding infection management of T-ALL patients, poor chemotherapy responses increased the unacceptably heightened risk induced by mixed infection. These patients would undergo a period of profound and prolonged immunosuppression so that opportunistic infections might occur. However, myelosuppression is more likely to contribute to febrile neutropenia and severe infections in the ETP subgroup. In this regard, ETP leukemia is defined by gene expression profiling and immunophenotype, representing a novel high-risk subtype of ALL.^(8,9,17,18)^ Our data showed that ETP leukemia had distinct clinical features presenting with significantly lower WBC and neutrophil count and reduced myeloid percentage at diagnosis when compared with other T-ALL. ETP leukemia is postulated to derive from a subset of early thymus-cell progenitor and transcriptionally related to hematopoietic stem cells and myeloid progenitors. [10, 24] It is reported that the earliest thymic T-cell progenitors sustain B and myeloid lineage potential and ETPs are major granulocyte precursors in mouse thymus [24]. We speculate that the origin of ETP leukemia harbors impairment in both T lymphopoiesis and myelopoiesis, resulting in persistent neutropenia. This hypothesis might partly explain more prolonged neutropenia in prediagnosis and induction in the ETP cohort than in the non-ETP cohort. In addition, high-frequency genomic mutation in ETP patients might be another potential factor for T lymphopoiesis and myelopoiesis [19]. Elucidating genetic profile and comparing the mutational genotypes of adult T-ALL might provide more valuable information in terms of the disease control and prevention. Unfortunately, we are not able to perform a comprehensive analysis due to lack of enough genomic mutation data on adult T-ALL in our center. Therefore, the exact relationship of persistent febrile neutropenia and long-term exposure to broad-spectrum antibiotics which induce drug resistance and mixed infection is uncertain, but this could be the reasons of the high prevalence of MDR and mixed infection in ETP-ALL.

Defining the potential predictors for infection will be valuable to reduce treatment-related toxicity. Previous studies have recognized Down syndrome, microbiome diversity, and severe neutropenia as preexisting risk factors for infection-associated adverse events [1–3, 9, 18, 26]. In this study, we identified myeloid percentage as a risk factor for mixed infection, with about 12.27% and 2.24% cut-off value defined by ROC analysis in the T-ALL and ETP-cohorts, indicating that the most of ETP ALL had a high risk of mixed infection during induction chemotherapy. These results also suggest us to reassess the intensive regimen during induction chemotherapy for T-ALL, particularly, for ETP-ALL. Because of the high incidence of infection-related complications in ETP patients, we recommended that more intensive monitoring and early antibiotic combination treatment should be applied. It is critical to the logistics management for ETP patients. To avoid severe infections, it is of importance that utilizing conventional and contemporary diagnostic tools identify pathogens and spectrum of infections to optimize antimicrobial therapy. Although being classified as a high risk with poor outcome, accumulating evidences indicate that pediatric-inspired protocols, such as Burlin-Frankfurt-Münster (BFM) backbone and GRALL-2003 Trials, have improved the outcome of ETP-ALL similar with other T-ALL [27]. Preliminary results of the PDT-ALL-LBL trial also showed promising outcomes for both ETP and other T-LBL/ALL. An optimal regimen for ETP-ALL might be based on reduced intensity of chemotherapy and reveal risk predictors to reduce induction-related infection and mortality to improve the outcome. [3, 4, 28–30]

In conclusion, our findings demonstrated that ETP ALL, a novel high-risk subtype, is more susceptible to induction-related infection. BM myeloid percentage is identified as a major risk factor for a poor outcome of infection-related complications. Due to limited sample size and lack of enough genomic mutation data, our dataset failed to identify more specific population(s) and other potential responsible risk factors. In addition, these results were just derived from a single-center data. A larger, multicenter, prospective cohort studies are needed to develop and validate an optimal scoring system for the risk of infection in the T-ALL and ALL populations.

## Figures and Tables

**Table 1:** Clinical characteristics of T-ALL adult patients.

Characteristics	ETP	Non-ETP	*P* value
	*n* = 46	*n* = 51	
Age	25 (19-33)	24 (17-32)	0.131
Median (IQR)			
Gender			
Female	15	12	
Male	31	39	0.442
Laboratory values			
Median (IQR)			
WBC count (×10^∗^9/L)	8.15 (3.13-66.07)	92.84 (13.36-187.29)	<0.001
Neutrophil count (×10^∗^9/L)	1.35 (0.73-5.03)	8.70 (4.13-14.38)	**<0.001**
Platelet count (×10^∗^9/L)	145 (76.76-198.5)	42 (24-147.5)	<0.001
Hemoglobin (g/dL)	102 (68.75-124.5)	116 (92.5-131.5)	0.117
LDH (IU/L)	270 (176.75-417.75)	1229 (425-2154.25)	<0.001
Duration of neutropenia	13 (9.25-18.75)	5 (0-10)	<0.001
BM meyloid percentage	13.35 (2.87-38.8)	35.31 (9.28-62.25)	0.007
Response to induction	*n* = 46	*n* = 51	
CR^∗^	21	40	0.001
PR/failure^∗^	25	11	**0.001**
Death in induction	4	3	0.593

Quantitative variables are expressed as median (IQR range). IQR: interquartile range; WBC: white blood cell; LDH: lactate dehydrogenase; BM: bone marrow; BM myeloid percentage: identified by flow cytometry and aspirate smear by Wright-Giemsa stain on bone marrow samples at diagnosis and measured as the mean plus or minus the standard deviation; CR^∗^: complete response after the first treatment; PR^∗^: partial response after the first treatment.

**(a):** 

Infection sites	Total	ETP	Non-ETP	*P*
Bloodstream	27	17	10	0.057
Pulmonary	9	8	1	0.01
Disseminated	23	19	4	
Crissum and mucosa	8	7	1	
Gastrointestinal tract	5	4	1	
Skin and soft tissue	5	4	1	
Central nervous system	2	1	1	
Tonsil	1	1	0	
Bone marrow	1	1	0	
Cardiovascular system	1	1	0	

**(b):** 

Causative agents	Total	ETP	Non-ETP	
	38 (100%)	27 (71.05)	11 (28.95)	
Gram-positive organisms	6 (15.79)	4 (10.53)	2 (5.26)	
*Staphylococcus*	3 (7.89)	2 (5.26)	2 (5.26)	
*Staphylococcus aureus*		1 (2.60)	1 (2.60)	
MRSA		1 (2.60)	0	
*Bacillus cereus*	2 (5.26)	1 (2.60)	1 (2.60)	
*Enterococcus faecium*	1 (2.60)	1 (2.60)	0	
Gram-negative organisms	26 (68.42)	18 (47.37)	8 (21.05)	
*Klebsiella* spp.	11 (28.95)	7 (18.42)	4 (10.53)	
*Klebsiella Pneumoniae*		4 (10.53)	3 (7.89)	
CRKP		1 (2.60)	0	
*Klebsiella ozaenae*		1 (2.60)	0	
Resistant *K. ozaenae*		1 (2.60)	0	
*Acinetobacter* spp.	2 (5.26)	2 (5.26)	0	
ACB complex		1 (2.60)	0	
PDRAB		1 (2.60)	0	
*Pseudomonas aeruginosa*	5 (13.16)	2 (5.26)	3 (7.89)	
*Escherichia*	3 (7.89)	3 (7.89)	0	
*Escherichia coli*		2 (5.26)	0	
ESBLs-producing *Escherichia coli*		1 (2.60)	0	
*Salmonella choleraesuis*	1 (2.60)	0	1 (2.60)	
*Aeromonas hydrophila*	2 (5.26)	1 (2.60)	1 (2.60)	
*Aeromonas hydrophila*		0	1 (2.60)	
Resistant *Aeromonas hydrophila*		1 (2.60)	0	
*Sphingomonas paucimobilis*	1 (2.60)	1 (2.60)	0	
*Stenotrophomonas maltophilia*	2 (5.26)	2 (5.26)	0	
Fungus	6 (15.79)	5 (13.16)	1 (2.60)	

MRSA: methicillin-resistant *Staphylococcus aureus*; CRKP: carbapenem-resistant *Klebsiella pneumoniae*, ACB complex: *A*. *calcoaceticus-A. baumannii* complex, PDRAB: pan-drug-resistant *Acinetobacter baumannii*.

**Table 3:** Univariate analysis identifying factors associated with IRM.

	T-ALL			ETP		
Causative agent	OR	95% CI	*P*	OR	95% CI	*P*
G-positive infection	1.929	0.203-18.36	0.568	4.222	0.33-54.085	0.268
G-negative infection	20.4	2.318-179.55	0.007	0	0	0.999
Fungal infection	0	0	0.999	0	0	0.999
Resistant infection	3.5	0.336-36.429	0.295	9.25	1.01-84.732	0.049
Mixed infection	10.25	1.852-56.736	0.008	21.6	1.866-250.025	0.014

G-negative MDI and mixed infection were critical risk factors associated with IRM in the T-ALL population. Mixed infection and resistant infection was identified factors associated with IRM in the ETP-ALL cohort. IRM: infection-related mortality.

**Table 4:** T-ALL and ETP ALL regression analyses of induction infection.

	T-ALL			ETP		
	OR	95% CI	*P*	OR	95% CI	*P*
Univariate regression analysis of mixed infection in the induction phase						
Characteristic						
WBC			0.555			0.819
WBC [1]	0.4	0.049-3.243	0.391	1.5	0.164-13.749	0.72
WBC [2]	1.071	0.15-7.642	0.945	1.929	0.25-14.887	0.529
NEU			0.323			0.843
NEU [1]	0.221	0.029-1.664	0.143	0.938	0.114-7.728	0.952
NEU [2]	0.5	0.054-4.672	0.543	0.556	0.059-5.241	0.608
BM myeloid percentage	0.975	0.94-1.01	0.156	0.915	0.843-0.993	0.033
CR	0.025	0.127-0.64	0.012	0.6	0.014-1.354	0.323

Multiple regression analysis of mixed infection in induction phase						
Characteristic						
WBC			0.146			0.388
WBC [1]	0.059	0.001-4.03	0.189	0.119	0.002-8.306	0.326
WBC [2]	2.25	0.072-69.965	0.644	1.434	0.045-45.853	0.838
NEU			0.823			0.512
NEU [1]	1.45	0.039-54.431	0.841	2.057	0.057-74.632	0.694
NEU [2]	0.472	0.012-19.311	0.692	0.211	0.003-13.712	0.465
BM myeloid percentage	0.895	0.807-0.992	0.035	0.864	0.75-0.995	0.043
CR	0.019	0.001-0.557	0.022	0.012	0-1.092	0.055

## Data Availability

The datasets during and/or analyzed during the current study are available from the corresponding author on reasonable request.

## Supplementary Materials

Supplementary Materials Supplemental Figure 1 Kaplan-Meier analysis of induction-related infections in ETP and non-ETP cohort. Supplemental Table 1: treatment protocol PDT-ALL-LBL. Supplemental Table 2: definitions related to infections. Supplemental Table 3: sites infections in prediagnosed phase. Supplemental Table 4: resistant infection characteristics of ETP patients. Supplemental Table 5: infection characteristics of infection-related mortality (IRM) T-ALL patients. Supplemental Table 6: T-ALL and ETP CDI risk factor regression analysis in prediagnosed phase.
