# Supplementary material for: Early T-Cell Precursor Leukemia Has a Higher Risk of Induction-Related Infection among T-Cell Acute Lymphoblastic Leukemia in Adult
Source: Mediators Inflamm. 2020 Dec 24;2020:8867760. doi: 10.1155/2020/8867760 (PMC7775137; doi:10.1155/2020/8867760)
Supplement: Supplementary Materials — Supplemental Figure 1 Kaplan-Meier analysis of induction-related infections in ETP and non-ETP cohort. Supplemental Table 1: treatment protocol PDT-ALL-LBL. Supplemental Table 2: definitions related to infections. Supplemental Table 3: sites infections in prediagnosed phase. Supplemental Table 4: resistant infection characteristics of ETP patients. Supplemental Table 5: infection characteristics of infection-related mortality (IRM) T-ALL patients. Supplemental Table 6: T-ALL and ETP CDI risk factor regression analysis in prediagnosed phase. [file 8867760.f1.docx]

**Supplemental Figure 1. Kaplan-Meier analysis of induction-related infections in ETP and non-ETP cohort.**

#
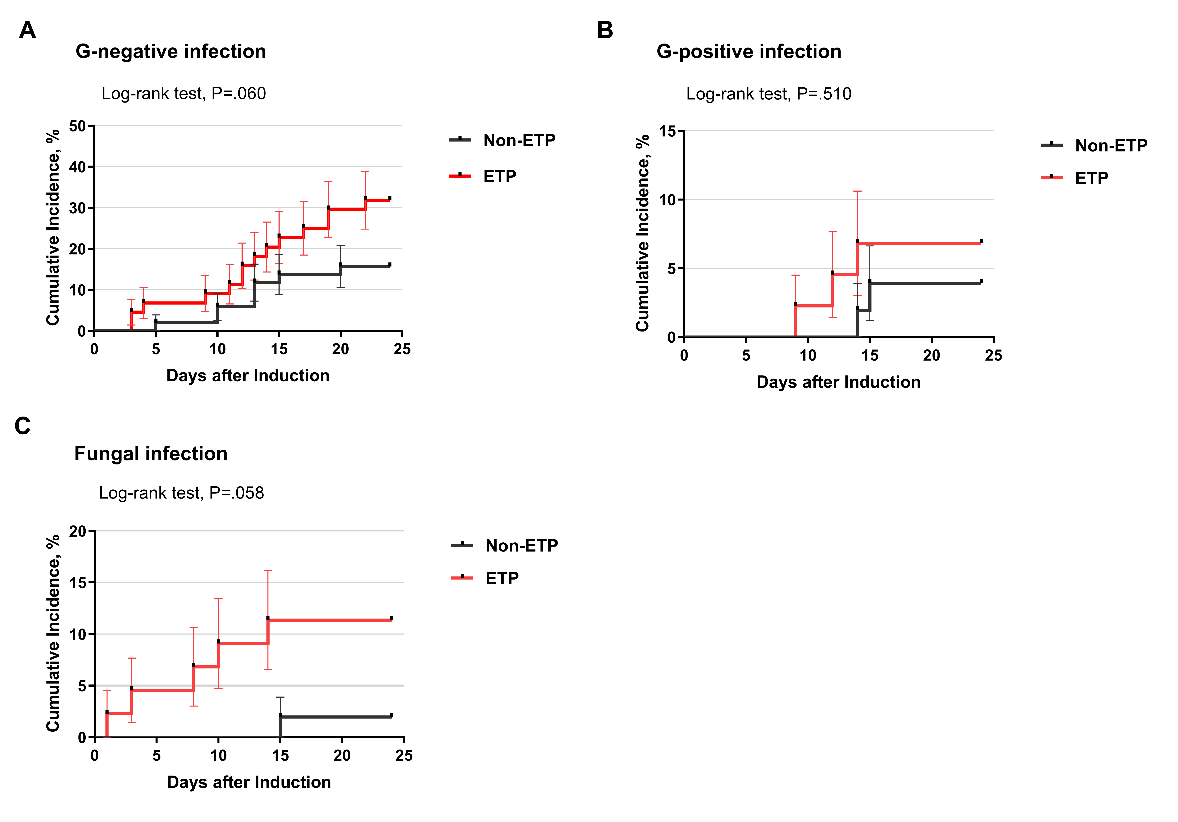


# Supplemental Table I. Treatment protocol PDT-ALL-LBL

| **Element** | **Drug** | **Dose** | **Given Day(s)** |
| --- | --- | --- | --- |
| **VICLD** | Vincristine/VCR  Idarubicin/IDA  Cyclophosphamide/CTX  Pegaspargase/PEG-asp  Dexamethasone  Chidamide | 1.4 mg/m^2^  10 mg/m^2^  1 g/m^2^  2000IU/m^2^  0.15mg/m^2^  10 mg | day 1, 8, 15, 22  day 1, 8  day 1  day 1,15  day 1 - 24  day 1 - 24 |

Bone marrow test and Flow-based MRD-assessment were performed on d15, d24 and d45 during induction phase.

**Supplemental Table 2. Definitions Related to Infections**

| **Outcome** | **Definition** |
| --- | --- |
| Neutropenia | Absolute neutrophil count ≤500/μL |
| Febrile neutropenia | Temperature >38.5 °C once or 38–38.5°C twice were taken within a 4-h interval. |
| Microbiologically documented infection (MDI) | Diagnosed bacterial, viral, fungal, or parasitic infection, with supportive microbiological evidence, such as a positive culture, antigen or PCR test results, or characteristic histopathological findings |
| Clinically documented infection (CDI) | Infection diagnosed by the treating clinician for which a specific microbial cause could not be demonstrated |
| Fever of unknown origin (FUO) | Fever of unknown origin was defined as a fever occurring in the absence of a positive microbiology result or clinical infection |
| Bloodstream infection | Any infection caused by a recognized pathogen that was isolated from ≥1 blood culture in the context of a compatible clinical illness; common commensal bacteria were included if identified from multiple culture sets, or if a single blood culture set was collected before start of antibiotic therapy and the result deemed clinically significant by the treating clinician |
| Mixed infection | Identified more than one microbiologically documented infection during first induction therapy |
| Infection-related mortality (IRM) | Any CDI/MDI related death during the induction treatment |

**Supplemental Table 3. Sites infections in pre-diagnosed phase**

| **Site of infection** | **Total** | **ETP** | **Non-ETP** |
| --- | --- | --- | --- |
| Pulmonary | 12 | 8 | 4 |
| Tonsil | 3 | 2 | 1 |
| Gastrointestinal tract | 2 | 2 | 0 |

**Supplemental Table 4. Resistant infection characteristics of ETP patients.**

| **Patients NO.** | **Gender** | **Age** | **Infection time after induction start** | **Type of pathogen** | **Site of resistant infection** | **CDI infeciton site** |
| --- | --- | --- | --- | --- | --- | --- |
| 1 | Male | 23 | 14 | MRSA | BM | Pulmonary，Crissumn |
| 2 | Female | 20 | 9 | PDRAB | Bloodstream | Pulmonary, Gastrointestinal tract |
|  |  |  | 9 | Resistant K.ozaenae | Bloodstream, central nervous system | Pulmonary, Gastrointestinal tract |
| 3 | Male | 25 | 1 | Resistant Aeromonas hydrophila | Bloodstream | Soft tissue |
| 4 | Male | 55 | 15 | ESBLs-producing Escherichia coli | Bloodstream | Crissumn |
| 5 | Male | 39 | 1 | CRKP | Pulmonary | Gastrointestinal tract |

**Supplemental Table5. Infection characteristics of infection-related mortality (IRM) T-ALL patients.**

| **Patients NO.** | **Subgroup** | **Gender** | **Age** | **Infection time after induction start** | **Pathogen** | **Infection site** | **Complication** | **Cause of death** |
| --- | --- | --- | --- | --- | --- | --- | --- | --- |
| 1 | ETP | 20 | Female | 9 | *Resistant K.ozaenae*，PDRAB | Blood | Peritonitis, intracranial infection，pneumonia,septic shock | Septic shock |
|  |  |  |  | 22 | PDRAB | Central nervous system | |  |
| 2 | ETP | 59 | Male | 10 | ACB complex，*Escherichia coli* | Blood | Respiratory failure，heart failure,septic shock | Respiratory failure,heart failure,septic shock |
| 3 | ETP | 30 | Male | / | / | / | Pneumonia，perianal infection,septic shock | Septic shock |
| 4 | ETP | 33 | Male | 12 | *Klebsiella Pneumoniae，Bacillus cereus* | | Infective endocarditis ,septic shock | Septic shock |
| 5 | Non-ETP | 26 | Male | 13 | *Aeromonas hydrophila* | Blood | Necrotizing fasciitis，septic shock | Septic shock |
| 6 | Non-ETP | 15 | Male | / | / | / | Septic shock | Septic shock |
| 7 | Non-ETP | 32 | Male | 20 | *Klebsiella Pneumoniae* | Blood,Central nervous system | Purulent meningitis，septic shock | Hernia cerebri,septic shock |

**Supplemental Table 6. T-ALL and ETP CDI risk factor regression analysis in pre-diagnosed phase**

| **Univariate regression analysis of CDI in pre-diagnosed phase** | | | | | | | |
| --- | --- | --- | --- | --- | --- | --- | --- |
|  | T-ALL | | |  | ETP | | |
|  | OR | 95% CI | P |  | OR | 95% CI | P |
| Characteristic |  |  |  |  |  |  |  |
| WBC |  |  | 0.464 |  |  |  | 0.734 |
| WBC(1) | 0.548 | 0.137-2.183 | 0.394 |  | 0.615 | 0.135-2.815 | 0.531 |
| WBC(2) | 0.36 | 0.069-1.88 | 0.226 |  | 0.533 | 0.096-2.976 | 0.474 |
| NEU |  |  | 0.054 |  |  |  | 0.277 |
| NEU(1) | 0.171 | 0.033-0.879 | 0.034 |  | 0.231 | 0.036-1.497 | 0.124 |
| NEU(2) | 0.625 | 0.105-3.707 | 0.605 |  | 0.625 | 0.105-3.707 | 0.605 |
| BM myeloid percentage | 0.922 | 0.874-0.973 | **0.003** |  | 0.894 | 0.821-0.972 | **0.009** |
| ETP | 2.635 | 0.733-9.472 | 0.138 |  |  |  |  |
|  |  |  |  |  |  |  |  |
| **Multiple regression analysis of CDI in pre-diagnosed phase** | | | | | | | |
|  | T-ALL | | |  | ETP | | |
|  | OR | 95% CI | P |  | OR | 95% CI | P |
| Characteristic |  |  |  |  |  |  |  |
| WBC |  |  | 0.956 |  |  |  | 0.506 |
| WBC(1) | 1.394 | 0.067-28.813 | 0.83 |  | 0.709 | 0.02-29.505 | 0.857 |
| WBC(2) | 1.482 | 0.11-19.911 | 0.767 |  | 0.193 | 0.01-5.691 | 0.341 |
| NEU |  |  | 0.205 |  |  |  | 0.643 |
| NEU(1) | 0.212 | 0.01-4.605 | 0.323 |  | 0.899 | 0.02-40.724 | 0.957 |
| NEU(2) | 3.052 | 0.191-48.804 | 0.43 |  | 3.096 | 0.12-80.514 | 0.497 |
| BM myeloid percentage | 0.901 | 0.837-0.969 | **0.005** |  | 0.859 | 0.76-0.966 | **0.011** |
| ETP | 0.873 | 0.101-7.543 | 0.901 |  |  |  |  |
